# Role of BIM Deletion Polymorphism and BIM Expression as Predictive Biomarkers to Maximize the Benefit of EGFR-TKI Treatment in EGFR-Positive NSCLC

**DOI:** 10.31557/APJCP.2019.20.12.3581

**Published:** 2019

**Authors:** Pimpin Incharoen, Chanchai Charonpongsuntorn, Sakditad Saowapa, Ekaphop Sirachainan, Thitiya Dejthevaporn, Kaettipong Kampreasart, Narumol Trachu, Dittapol Muntham, Thanyanan Reungwetwattana

**Affiliations:** 1 *Department of Pathology, *; 2 *Division of Medical Oncology, Department of Internal Medicine, *; 4 *Reasearch Center, Faculty of Medicine, Ramathibodi Hospital, Mahidol University, *; 3 *Faculty of Medicine, Srinakharinwirot University, Bangkok, *; 5 *Department of Mathematics, Faculty of Science and Technology, Rajamangala University of Technology Suvarnabhumi, Thailand. *

**Keywords:** BCL2L11, BH3 protein, pro-apoptotic, anti-apoptotic

## Abstract

**Objective:**

BIM is a modulator of apoptosis that is triggered by EGFR-TKIs. This study evaluated the role of BIM deletion and its expression as predictor of EGFR-TKI treatment outcome.

**Methods:**

The medical record of 185 EGFR-positive advanced non-small cell lung cancer (NSCLC) patients with/ without EGFR-TKI treatment between 9/2012 and 12/2014 were retrospectively reviewed. BIM deletion polymorphism and expression were tested by RT-PCR and immunohistochemistry, respectively. Survival outcomes in EGFR-TKI-treated patients were analyzed according to treatment sequence and EGFR mutation. The correlation between BIM deletion polymorphism, expression, response rate (as a function of EGFR-TKI treatment) and schedule was also explored.

**Result:**

EGFR-TKIs were administered to 139 (75.1%) of the 185 patients: as the first-line in 52 (37.4%) patients and as later-line treatment in 87 (62.6%) patients. Median overall survival (mOS) was significantly longer in EGFR-TKIs treated patients (28.9 vs. 7.4 months, P<0.001). Among L858R-mutated patients, median progression-free survival (mPFS) was significantly longer in first-line EGFR TKI treatment than a later-line (12.6 vs. 6.3 months, P=0.03). BIM deletion polymorphism and expression was detected in 20.2% and 52.7%, respectively. Patients without BIM deletion polymorphism had a significantly longer mOS when treated with a first-line than with a later-line EGFR-TKI (28.9 vs. 20.7 months, P= 0.04). Patients without BIM expression had a significantly longer mPFS (9.6 vs. 7.3 months, P=0.01) better mOS and response rate (RR).

**Conclusion:**

BIM deletion polymorphism and expression may predict an EGFR-TKI response in patients with EGFR-positive during therapy.

## Introduction

Lung cancer is one of the most common causes of malignancy-related death in the world, and most cases are non-small cell lung cancer (NSCLC) (Goldstraw et al., 2011; Jemal et al., 2011; Siegel et al., 2013). In NSCLC, systemic chemotherapy is the standard treatment for advanced disease. An activating mutation of the epidermal growth factor receptor (*EGFR*) gene is a common driver of NSCLC (Lynch et al., 2004). The frequency of EGFR mutation depends on the population, with a high frequency (30%–50%) in East Asia, including Thailand (Shigematsu et al., 2005). EGFR tyrosine kinase inhibitors (TKIs), such as erlotinib, gefitinib and afatinib, have produced dramatic responses in patients with EGFR-positive NSCLC, as evidenced by significant improvements in the response rate (RR) and survival compared with patients treated with platinum doublet-based chemotherapy as first-line therapy. Moreover, EGFR-TKIs have also improved the quality of life (Han et al., 2012; Maemondo et al., 2010; Mitsudomi et al., 2010; Mok et al., 2009; Yang et al., 2015; Zhou et al,. 2011) Currently, EGFR-TKI treatment is the standard first-line therapy for patients with NSCLC characterized by activating EGFR mutations. However, whether sequence EGFR-TKI should be given as the first- or later-line treatment remains controversial.

Approximately 30% of EGFR-positive NSCLCs do not respond to EGFR-TKIs (Goldstraw et al., 2011; Lynch et al., 2004; Shigematsu et al., 2005) but the mechanism of intrinsic resistance is poorly understood. Recently, a role for BIM deletion polymorphism as a mechanism of intrinsic EGFR-TKI resistance was reported (Lee et al., 2014; Ng et al., 2012; Zhao et al., 2014). BIM, also known as B-cell chronic lymphocytic leukemia/lymphoma (Bcl-2)-like 11 (BCL2L11), is a member of the Bcl-2 family gene. BIM encodes the BH3 protein, which activates cell death either by opposing the pro-survival activities of members of the BCL2 family or by binding to and directly activating pro-apoptotic BCL2 family members (Youle and Strasser, 2008; Akiyama et al., 2009). In EGFR-mutant lung cancer, BIM has a role in inducing cellular apoptosis after EGFR TKIs treatment (Gong et al., 2007). A common intronic deletion polymorphism in the gene encoding BIM has been described in which BIM splicing is switched from exon 4 to exon 3 resulting BIM isoforms lack pro-apoptotic BH3 activity causing resistant of EGFR TKIs in NSCLC cell line (Ng et al., 2012). Patients with EGFR-positive NSCLC characterized by a confirmed BIM deletion polymorphism also have a shorter PFS (Isobe et al., 2014; Lee et al., 2014; Zhao et al., 2014).

Reimbursement for EGFR-TKI therapy differs from country to country. In low- to middle-income countries, such as Thailand, the cost of EGFR-TKI is reimbursed by the Civil Servant Medical Benefit Scheme (CSMBS) only when these drugs are prescribed as a second- or later-line therapy; otherwise, patients have to pay out-of-pocket. It is therefore important to establish the optimal sequence of EGFR-TKI therapy in patients with EGFR-positive NSCLC. 

The aim of the present study is to explore BIM polymorphism and BIM expression status in EGFR-positive NSCLC patient to be used as the prognostic marker for the treatment outcome of EGFR-TKI therapy and to identified patients who will most benefit from EGFR-TKI treatment and may thus be eligible for the reimbursement of treatment costs.

## Materials and Methods

*Patient population and clinical data collection*

This retrospective study included 185 patients with recurrent or stage IIIB or IV EGFR-positive NSCLC who were diagnosed and treated between September 1, 2012 and December 31, 2014. The patients were identified from a pathological and cancer registry database. Clinical data, including age, sex, smoking status, body mass index, performance status, pathological diagnosis, type of EGFR mutation, metastasis, treatment, and recurrence pattern, were collected systematically from our department’s electronic database. Disease staging was conducted according to the seventh edition of the TNM classification system. Among the 185 patients, 139 received at least one EGFR-TKI during disease therapy.

PFS was calculated from the date of first systemic therapy administration to the date of disease progression, unacceptable toxicity or death from any cause. OS was defined as the date from disease diagnosis or recurrence to the date of either death from any cause or the last follow-up. ORR was reviewed based on the data and an analysis of related images according to the Response Evaluation Criteria in Solid Tumors (RECIST) criteria, version 1.1. 

This single-center study was conducted at Ramathibodi Hospital, Mahidol University (Bangkok, Thailand) and was approved by the Ramathibodi Research Ethical Committee. 

*Analysis of the tumor samples*

The detection of a BIM deletion polymorphism from formalin-fixed, paraffin-embedded (FFPE) tissues by real-time polymerase chain reaction (RT-PCR) was possible for 129 patients, while BIM expression was confirmed by immunohistochemistry (IHC) in samples from 131 patients. 

**Figure 1:**
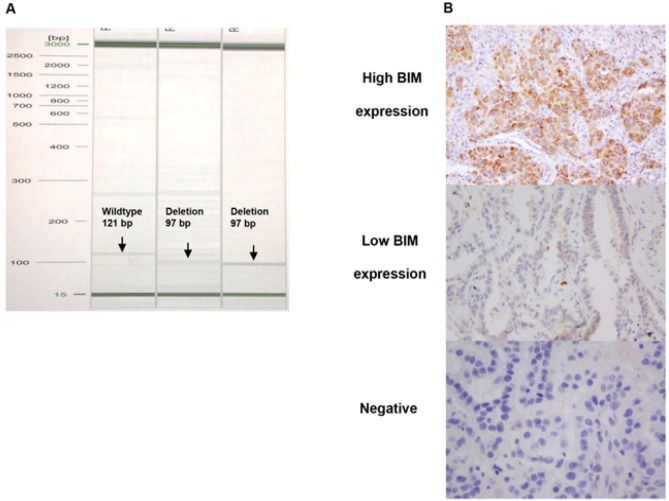
BIM Deletion Polymorphism and Expression by RT-PCR and Immunohistochemistry Staining. Notes: (A) Agarose gel electrophoresis of BIM deletion polymorphism and wild-type, (B) IHC for Anti-BIM (200x)

DNA extracted from FFPE was subjected to PCR amplification using primers designed to detect the deletion site (2903 bp) in intron 2 of the BCL2L11 gene. Two separate primer sets were designed for the detection of wild-type and deletion alleles. The forward and reverse primers for the wild-type allele were 5′CAGTGAGGTAAATCAGGCAGGC3′ and 5′ATGTCTGTCATTTCTCCCCACC3′, respectively. The forward and reverse primers for the deletion allele were 5′AGGCTTCAGTGAGGTAAATCACTGT3′ and 5′TGGTAAGTATGTGGAGAAACTGGAAC3′, respectively. The PCR products for the deletion (97 bp) and wild-type (121 bp) alleles were analyzed by agarose gel electrophoresis (Figure 1).

Anti-BIM or anti-BCL-2 antibodies targeting the BCL-2 homology domain 3 (BH3) were used in the IHC analyses. Histological sections (4 μm thick) were prepared from the FFPE blocks and incubated in 10 mM citrate buffer, pH 6.0 for 20 min at 121°C in an autoclave to retrieve the antigen. Endogenous peroxidase activity was blocked by immersing the sections for 20 min in methanol containing 1.5% hydrogen peroxide, followed by incubation with normal rabbit serum to block nonspecific antibody-binding sites. The sections were incubated overnight at 4°C with BIM rabbit polyclonal antibody (K.912.7, Thermo Fisher Scientific, USA) diluted 1:100. IHC BIM staining levels were scored as high, low, or negative as previously described (Berrieman et al., 2005; Borner et al., 1999). Moderate to strong staining intensity in > 50% of the tumor cells was defined as high BIM expression and in < 50% as low BIM expression (Figure 1).

*Statistical analysis*

Statistical analyses were performed using STATA version 14.1 (Stata Corp, College Station, Texas, USA). All categorical variables were compared with χ2 tests or Fisher’s exact test. Median progression-free survival (mPFS) and median overall survival (mOS) were calculated using the Kaplan-Meier method, and differences were compared using the log-rank test. Differences in the overall response rate (ORR) between patients with or without EGFR-TKI therapy and with respect to BIM deletion polymorphism and expression status were compared using Fisher’s exact test. Univariate analysis and multivariate Cox regression analysis were performed to identify factors associated with shorter PFS and OS. The hazard ratio (HR) and 95% confidence interval (CI) were estimated. A P-value <0.05 was considered to indicate statistical significance. 

## Results

*Patient characteristics*

The study enrolled 185 patients with a diagnosis of advanced or recurrent EGFR-positive NSCLC who visited Ramathibodi Hospital between September 1, 2012 and December 31, 2014. The median follow-up time was 17.4 months. The cut-off for data collection was October 31, 2015. The baseline characteristics of the patients according to EGFR-TKI treatment are presented in Table 1. Among the 185 patients with EGFR-positive NSCLC, 139 (75.1%) received EGFR-TKI therapy at any time point during treatment and 46 (24.9%) did not. Patients who received EGFR-TKI treatment at any time point, including those who received multiple lines of treatment, had a significantly better ECOG performance status (Table 1). OS was significantly longer in patients treated with an EGFR-TKI at any time point than in patients not treated with EGFR-TKI (28.9 vs. 7.4 months, (HR = 0.25 [95% CI = 0.16–0.40], P<0.001)

*Results according to the sequence of EGFR-TKI therapy*

EGFR-TKI therapy (erlotinib or gefitinib) was administered as first-line treatment to 52 of the 139 patients (37.4%) and as a later-line treatment to 87 patients (62.6%). Patients who received first-line EGFR-TKI treatment had a longer, but not statistically significant, mPFS than patients in the later-line treatment group (9.2 vs. 8.2 months, P=0.26). There was no significant difference in the mOS (Table 2). Patients with a L8585R-positive tumor had a significantly better mPFS (12.6 vs. 6.3 months, P = 0.03) and a trend of a longer mOS (28.9 vs. 25.6 months) if EGFR-TKI was the first-line rather than the later-line treatment. Patients with rare EGFR mutations, including G719X, exon 20 mutation or a de novo T790M mutation, had a worse PFS (2.5 months) than patients with other EGFR mutation types regardless of the sequence of EGFR-TKI treatment. There were no significant differences in the mOS and overall response rate (ORR) of the first-line and later-line EGFR-TKI treatment groups (Table 2). However, among patients with L858R-mutation-positive NSCLC, the ORR was better in the first-line than in the later-line treatment group (78.3% vs. 48.5%, P=0.08) (Table 2).

*Detection of BIM deletion polymorphism and BIM expression in EGFR-positive NSCLC*

BIM deletion polymorphism was assessed by RT-PCR in 129 patients and by IHC in 131 patients with available archival tissue blocks. BIM deletion polymorphism was present in 26 of the 129 patients (20.2%), with a homozygous deletion in one patient and heterozygous deletions in the remaining 128 patients. IHC revealed BIM positivity in 69 of the 131 patients (52.7%), including high-level expression (>50% positive tumor cells) in 8 patients (Figure 1).

*The efficacy of EGFR-TKI treatment as a function of BIM deletion polymorphism and BIM expression*

**Figure 2:**
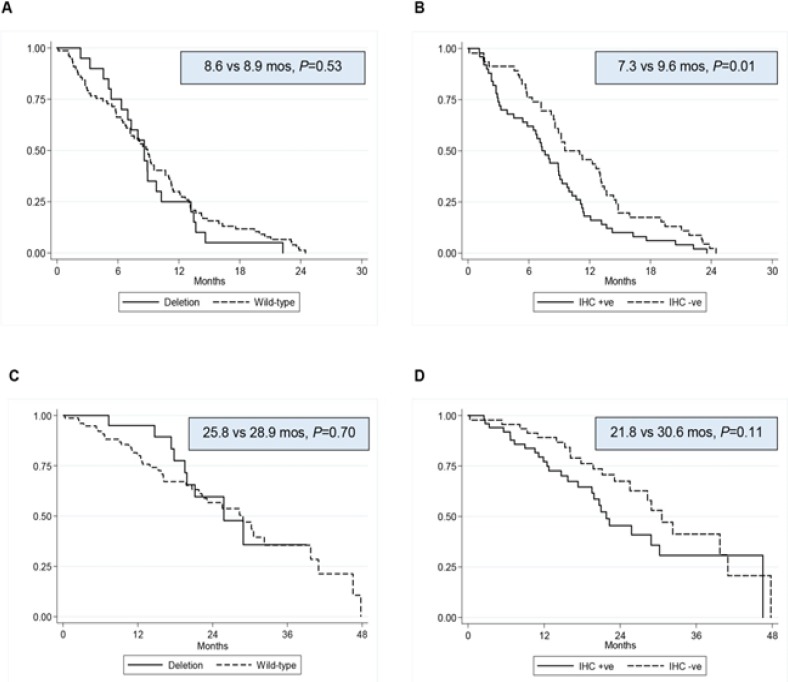
Kaplan-Meier survival curves of patients with EGFR-mutation-positive advanced NSCLC who received EGFR-TKI treatment, evaluated with respect to BIM deletion polymorphism and BIM expression status. Notes: PFS was assessed in patients with EGFR-positive NSCLC characterized by BIM deletion polymorphism vs. wild-type (A), and by positive vs. negative BIM expression (B). OS was categorized by BIM deletion polymorphism vs. wild-type (C) and by positive vs. negative BIM expression (D).

Among the 139 patients with EGFR-mutation-positive advanced NSCLC who received EGFR-TKI treatment, 97 had available FFPE tissue that could be analyzed for BIM deletion polymorphism and 96 had tissue samples allowing an analysis of BIM expression. The baseline characteristics of these patients are shown in Table 3. The clinical characteristics of patients with a BIM deletion polymorphism did not significantly differ from those with wild-type BIM. The mPFS and ORR were similar between the two groups, whereas patients with wild-type BIM had a trend of better mOS (Table 4). BIM expression was evaluated by IHC staining in 96 patients treated with EGFR-TKI. Those with BIM-expression-positive disease had a significantly worse mPFS (7.3 vs. 9.6 months, P=0.01) and a trend of a worse mOS (21.8 vs. 30.6 months, P=0.11), as shown in Table 4 and Figure 2.

*BIM deletion polymorphism and BIM expression as biomarkers predictive of the response to EGFR-TKI treatment*

Patients with BIM deletion polymorphism or positive BIM expression had a trend of a worse survival outcome (Table 5). A subgroup analysis in which the sequence of EGFR-TKI treatment was stratified showed a significantly better mOS in patients with BIM-expression-negative NSCLC (20.7 vs. 28.9 months, P=0.04). Patients who received later-line EGFR-TKI therapy for L858R-mutation-positive NSCLC characterized by BIM deletion polymorphism had a significant shorter PFS than did patient with wild-type BIM tumors (3.2 vs. 6.7 months, P=0.04). 

In a univariate Cox regression analysis of the factors predictive of OS and PFS in patients with EGFR-mutation-positive NSCLC treated with EGFR-TKI, IHC-detected BIM expression was the only predictive factor for both mOS (HR=1.66, 95% CI=1.010122.72, P 0.04) and mPFS (HR=1.69, 95% CI=1.12–2.55, P=0.01) (Table 6).

**Table 1:** Baseline Characteristics of Patients with Advanced EGFR-Positive NSCLC with or without EGFR-TKI Treatment

Characteristics	With EGFR-TKI treatment (N=139; 75.1%)	Without EGFR-TKI treatment (N=46; 24.9%)	*P-value*
Age			
< 65	78 (56.1)	24 (52.2)	0.64
≥ 65	61 (43.9)	22 (47.8)	
Sex			
Male	53 (38.1)	24 (52.2)	0.09
Female	86 (61.9)	22 (47.8)	
ECOG			
0-1	113 (81.3)	31 (67.4)	0.05
>1	26 (18.7)	15 (32.6)	
Smoking status			
Non-smoker	109 (78.4)	35 (76.1)	0.74
Ex-smoker	30 (21.6)	11 (23.9)	
Staging			
Recurrent	29 (20.9)	10 (21.7)	0.9
Metastasis	110 (79.1)	36 (78.3)	
Number of metastatic site			
≤ 2	89 (80.9)	28 (84.8)	0.61
> 2	21 (19.1)	5 (15.2)	
EGFR mutation status			
Exon 19 deletion	66 (47.5)	25 (54.3)	0.86
Exon 21 L858R mutation	56 (40.3)	17 (37.0)	
Combined mutation	7 (5.0)	1 (2.2)	
Rare mutation	10 (7.2)	3 (6.5)	
Brain metastasis			
No brain metastasis	111 (79.9)	39 (84.8)	0.46
Brain metastasis	28 (20.1)	7 (15.2)	
Number of systemic treatments			
≤ 2	83 (59.7)	22 (95.7)	<0.01
> 2	56 (40.3)	1 (4.3)	

Notes, The data are reported as the number (%).

**Table 2:** Efficacy of First- vs. Later-Line EGFR-TKI Treatment in Patients with Advanced EGFR-Positive NSCLC

	EGFR-TKI treatment (N = 139)		
	First line EGFR-TKI (N = 52 patients)	Later-line EGFR-TKI (N = 87 patients)	*P-value*
mPFS, months, N (%)			
Overall mutants	9.2 (52)	8.2 (87)	0.26
19 deletion	8.9 (21)	9.2 (45)	0.97
L858R mutation	12.6 (22)	6.3 (33)	0.03
Rare EGFR mutation	2.5 (8)	2.5 (9)	0.92
mOS , months, N (%)			
Overall mutant	23.1 (52)	32.3 (87)	0.3
19 deletion	28.9 (21)	39.8 (45)	0.14
L858R mutation	28.9 (22)	25.6 (33)	0.85
Rare EGFR mutation	18.8 (8)	41.0 (9)	0.4
Overall response rate			
Overall mutants, N (%)			
CR/PR	32 (61.5)	43 (49.4)	0.38
SD	15(28.8)	30 (34.5)	
PD	5 (9.6)	14 (16.1)	
19 deletion, N (%)			
CR/PR	13 (61.9)	25 (55.6)	0.93
SD	6 (28.6)	15 (33.3)	
PD	2 (9.5)	5 (11.1)	
L858R mutation, N (%)			
CR/PR	18 (78.3)	16 (48.5)	0.08
SD	4 (17.4)	13 (39.4)	
PD	1 (4.3)	4 (12.1)	
Rare mutations, N (%)			
CR/PR	1 (12.5)	2 (22.2)	0.37
SD	5 (62.5)	2 (22.2)	
PD	2 (25)	5 (55.6)	

**Table 3:** Baseline Clinical Characteristics with Respect to BIM Deletion Polymorphism Status in Patients with EGFR-Positive Advanced NSCLC

	BIM deletion (N=20 patients)	BIM wild type (N=77 patients)	*P-* *value*
Age, N (%)			
< 65	11 (55)	49 (63.6)	0.48
≥ 65	9 (45)	28 (36.4)	
Sex, N (%)			
Male	7 (35)	27 (35.1)	1
Female	13 (65)	50 (64.9)	
ECOG, N (%)			
0-1	16 (80)	60 (77.9)	1
>1	4 (20)	17 (22.1)	
Smoking, N (%)			
Non-smoker	15 (75)	65 (84.4)	0.32
Ex-smoker	5 (25)	12 (15.6)	
Staging, N (%)			
Recurrent	3 (15)	20 (26)	0.3
Metastasis	17 (85)	57 (74)	
EGFR mutation, N (%)			
Exon 19 deletion	11 (55)	36 (46.8)	0.31
L858R mutation	9 (45)	29 (37.7)	
Combined mutation	0	6 (7.8)	
Rare mutations	0	6 (7.8)	
Brain metastasis, N (%)			
Brain metastasis	3 (15)	17 (22.1)	0.49
No brain metastasis	17 (85)	60 (77.9)	
Number of systemic treatment, N (%)			
≤ 2	11 (55)	45 (58.4)	0.78
> 2	9 (45)	32 (41.6)	
BIM expression^a^, N (%)			
Positive	10 (52.6)	38 (53.5)	0.95
Negative	9 (47.4)	33 (46.5)	

Notes, ^a^N, 90

**Table 4:** Summary of EGFR-TKI Treatment Response in Patients with EGFR-Positive Advanced NSCLC Characterized by BIM Deletion Polymorphism or BIM Expression

	BIM deletion polymorphism			BIM expression		
	Mutant	Wild-type	*P-value*	Positive	Negative	*P-value*
mPFS, no. of months (%)						
Overall mutants	8.6 (20)	8.9 (77)	0.53	7.3 (50)	9.6 (46)	0.01
19 deletion	8.6 (11)	8.8 (36)	0.56	8.0 (21)	8.9 (25)	0.36
L858R mutation	8.6 (9)	9.3 (29)	0.47	9.0 (20)	12.6 (18)	0.27
mOS, no. of months (%)						
Overall mutants	25.8 (20)	28.9 (77)	0.7	21.8 (50)	30.6 (46)	0.11
19 deletion	25.8 (11)	39.8 (36)	0.55	25.8 (21)	32.3 (25)	0.36
L858R mutation	ND (9)	22.3 (29)	0.56	21.8 (20)	25.6 (18)	0.34
Overall response rate						
Overall mutants, N (%)						
CR/PR	13 (65)	41 (53.2)	0.73	23 (46)	31 (67.4)	0.06
SD	5 (25)	24 (31.2)		17 (34)	12 (26.1)	
PD	2 (10)	12 (15.6)		10 (20)	3 (6.5)	
19 deletion, N (%)						
CR/PR	7 (63.6)	19 (55.3)	0.62	9 (42.9)	18 (72)	0.14
SD	4 (36.4)	12 (33.3)		9 (42.9)	6 (24)	
PD	0	5 (13.9)		3 (14.3)	1 (4)	
L858R mutation, N (%)						
CR/PR	6 (66.7)	19 (65.5)	0.55	13 (65)	11 (61.1)	0.29
SD	1 (11.1)	7 (24.1)		3 (15)	6 (33.3)	
PD	2 (22.2)	3 (10.3)		4 (20)	1 (5.6)	

ND, not determined

**Table 5:** Subgroup Analysis of PFS and OS as a Function of BIM Status and Depending on the Mutation and Sequence of EGFR-TKI Treatment

	BIM deletion polymorphism			BIM expression		
	Mutant	Wild-type	*P-value*	Positive	Negative	*P-value*
	(N=20 patients)	(N=77 patients)		(N=50 patients)	(N=46 patients)	
mPFS, no. of months (%)						
First line	8.9 (8)	9.2 (34)	0.55	7.2 (21)	12.6 (20)	0.06
19 deletion	8.6 (3)	8.8 (14)	0.32	7.2 (6)	8.6 (10)	0.64
L858R	13.2 (5)	12.6 (13)	0.65	9.8 (9)	13.0 (9)	0.52
Later line	7.0 (12)	8.2 (43)	0.69	7.6 (29)	8.9 (26)	0.18
19 deletion	8.0 (8)	8.2 (22)	0.53	8.0 (15)	8.9 (15)	0.65
L858R	3.2 (4)	6.7 (16)	0.04	6.7 (11)	9.6 (9)	0.64
mOS, no. of months						
First-line	21.2	23.1	0.97	20.7	28.9	0.04
19 deletion	28.9	30.6	0.62	19.6	28.9	0.61
L858R	21.2	22.3	0.99	21	21.2	0.56
Later-line	25.8	30.3	0.72	25.8	39.8	0.37
19 deletion	25.8	39.8	0.6	25.8	39.8	0.31
L858R	NR	25.6	0.44	21.8	28.3	0.54

**Table 6:** Univariate Analysis of Factors Predicting PFS and OS in Patients with EGFR-Positive NSCLC who Received EGFR-TKI

Variable	PFS		OS	
	HR (95% CI)	P-value	HR (95% CI)	P-value
Age				
< 65 vs. ≥ 65	0.74 (0.53–1.04)	0.09	1.22 (0.82–1.84)	0.33
ECOG				
≥ 2 vs. < 2	1.44 (0.93–2.22)	0.1	3.84 (2.48–5.92)	< 0.01
Smoking				
Non-smoker vs. smoker	0.89 (0.59–1.34)	0.58	0.98 (0.60–1.59)	0.93
Sex				
Male vs. female	1.19 (0.84–1.67)	0.36	0.79 (0.53–1.19)	0.26
Brain metastasis				
Yes vs No	0.95 (0.63–1.45)	0.83	1.08 (0.66–1.77)	0.75
EGFR mutation				
Exon 19 deletion				
L8585R mutation	1.17 (0.81–1.69)	0.85	1.00 (0.65–1.54)	0.99
Other mutations	1.59 (0.92–2.72)	0.1	1.16 (0.61–2.19)	0.65
BIM deletion polymorphism				
Deletion vs. wild-type	1.17 (0.71–1.93)	0.63	0.86 (0.46–1.62)	0.64
BIM expression				
Positive vs. negative	1.69 (1.12–2.55)	0.01	1.66 (1.01–2.72)	0.04

## Discussion

First-line EGFR-TKI treatment is the standard treatment for patients with EGFR-positive advanced NSCLC. Randomized controlled trials and meta-analyses have confirmed a longer PFS and higher ORR in biomarker-selected patients who received EGFR-TKI therapy rather than platinum-based doublet chemotherapy (Han et al., 2012; Maemondo et al., 2010; Mitsudomi et al., 2010; Mok et al., 2009; Yang et al., 2015; Zhou et al,. 2011). However, the difference in the OS of patients treated with EGFR-TKI vs. chemotherapy was not statistically significant, due to crossover effects in each trial. Recently, a significant benefit in OS was determined in a subgroup analysis of patients with NSCLCs classified based on del19 mutations in Lux-lung 3 and Lux-lung-6 (Yang et al., 2015).

Most patients in low- to middle-income countries do not have access to EGFR-TKIs. In Thailand, there are also issues related to reimbursement for this mode of therapy in patients with EGFR-positive NSCLC, since the CSMBS does not cover the cost of EGFR-TKI for first-line treatment, only second- or later-line treatment. Patients who opt for first-line treatment have to pay out-of-pocket. Furthermore, there have been few randomized controlled trials comparing first- and second-line EGFR-TKI treatment. The TORCH study is the only randomized study that compared first-line EGFR-TKI followed by chemotherapy vs. first-line chemotherapy followed by second-line EGFR-TKI (Gridelli et al., 2012). The results showed that first-line erlotinib followed by cisplatin/gemcitabine was significantly inferior in terms of mOS. However, the population was non-selected and biomarker testing was available for only 39 patients. In addition, the interpretation of treatment outcome was confounded by the small sample size, including the <60% of patients who received the planned second-line therapy.

Our retrospective analysis demonstrated a s significantly longer mOS in patients with EGFR-positive NSCLC who received EGFR-TKI treatment (regardless of the treatment sequence) than in those who did not (28.9 vs. 7.4 months, P<0.001). This result was similar to those previously reported (21.6–35.5 months) (Han et al., 2012; Maemondo et al., 2010; Mitsudomi et al., 2010; Mok et al., 2009; Yang et al., 2015; Zhou et al,. 2011).

Our study also explored whether the sequence of EGFR-TKI therapy, first- or later-line, had an impact on survival outcome. While there was no statistically significant difference in terms of mPFS, mOS, and ORR in patients with NSCLC who received first- vs. later-line EGFR-TKI treatment, regardless of EGFR mutation type, patients with EGFR-positive NSCLC characterized by L858R mutation had a significantly longer mPFS if they received EGFR-TKI as the first-line treatment (12.6 vs. 6.3 months, P=0.03). Regarding the previous studies, several studies have reported that patients with NSCL carrying an exon 19 deletion had a better OS and better PFS than did patients with L858R-mutation-positive NSCLC (Goto et al., 2013; Jackman et al., 2006; Riely et al., 2006). Zhang et al., (2014) performed a meta-analysis of 13 studies of EGFR-positive (either exon 19 or 21) advanced NSCLC in which patients received first-line EGFR-TKI. The pooled hazard ratio of EGFR-TKI/chemotherapy for PFS was 0.28 (95% CI=0.20–0.38, P<0.001) in patients with NSCLC positive for exon 19 deletion and 0.47 (95% CI=0.35–0.64, P<0.001) in those with NSCLC positive for exon 21 L858R mutation. This result indicates a difference in the biology of NSCLCs carrying exon 19 deletions vs. exon 21 mutation. One of the rationale showed it might be because of the binding between the EGFR TKIs and ATP binding pocket in EGFR exon 19 deletion is stronger than EGFR exon 21 L858R. Regarding all previous information and our study’s result, we think if we could treat L858R mutant patients with EGFR TKIs as the first-line treatment then the survival outcome will be significantly better than if we wait and treat them with late-line EGFR TKIs. This will be one of the important guidance for oncologists in low- and middle-income countries in which health economics influence treatment choices.

The present study examined the role of BIM deletion polymorphism and BIM expression as predictors of the response to EGFR-TKI treatment. The 20.2% (26/129) prevalence of BIM deletion polymorphisms in our cohort was comparable to that in other studies of Asian populations (Isobe et al., 2014; Lee et al., 2014; Zhao et al., 2014). There were no significant differences between clinical characteristics, mPFS, mOS, and ORR to EGFR-TKI treatment among patients with or without NSCLC positive for BIM deletion polymorphism. However, patients with BIM-deletion-polymorphism positive NSCLC had a shorter mPFS and mOS than did patients with NSCLC carrying the wild-type BIM. Furthermore, patients with L858R mutation-positive NSCLC and wild-type BIM had a significantly better mPFS if they received EGFR-TKI as the later-line therapy. A previous retrospective and meta-analysis also showed that the presence of a BIM deletion polymorphism predicted a poor response to EGFR-TKI treatment (Ma et al., 2015; Ying et al., 2015), similar to our findings.

We also found that mPFS was a significantly longer in patients whose tumors were negative for BIM expression than in those whose tumors had sensitive EGFR mutations. The former also had significantly longer mOS when EGFR-TKI was the first-line treatment. The univariate analysis identified BIM expression as a strong and significant predictive biomarker for EGFR-TKI response. By contrast, Faber et al., (2011) reported a poor PFS (4.7 vs. 13.7 months, P=0.007) among patients with low BIM-RNA-expressing NSCLC, which correlated with low BIM protein expression on IHC staining. However, IHC staining for BIM expression using a BCL-2 antibody might not detect all anti-apoptotic pathways. In addition, the BIM pathway might not be the only protein critical in the apoptotic pathway, as other apoptotic regulators may also be essential. These hypotheses remain to be explored in further studies.

BIM-associated resistance to EGFR-TKIs may be surmountable with targeted therapies, such as the BH3-mimetic drug ABT-737, which enhance apoptotic signaling and cell death, as well as with histone deacetylase inhibitors (Nakagawa et al., 2013). The addition of these novel drugs to an EGFR-TKI regimen may prolong PFS and OS in patients with NSCLC carrying a BIM deletion polymorphism. Again, further studies are needed to determine the clinical efficacy of this approach to therapy.

This study had several limitations. Firstly, the sample size in no-EGFR-TKI treatment was small. Secondly, as discussed above, there is no standardized antibody and scoring system for BIM IHC staining. Last, we did not examine mRNA expression nor did we test the functionality of BIM, due to limited available tumor tissue.

In conclusions, EGFR-TKI efficiently targets EGFR-positive NSCLC. However, treatment reimbursement is an important issue in low- and middle-income countries. Our study demonstrated that BIM deletion polymorphism and BIM expression may serve as biomarkers predictive of an EGFR-TKI response. Confirmation of this finding would aid in selecting patients likely to greatly benefit from EGFR-TKI and thus be eligible for a reimbursement of treatment costs. Patients with L858R-positive NSCLC should receive EGFR-TKI as the first-line treatment to improve survival. Our results merit further study in a larger cohort.
